# *N*-Benzoyl-*N*-(1,4-dioxonaphthalen-2-yl)benzamide

**DOI:** 10.1107/S1600536812030231

**Published:** 2012-07-07

**Authors:** Yakini Brandy, Ray J. Butcher, Oladapo Bakare

**Affiliations:** a Department of Chemistry, Howard University, 525 College Street NW, Washington, DC 20059, USA

## Abstract

The title mol­ecule, C_24_H_15_NO_4_, crystallizes with two mol­ecules in the asymmetric unit (*Z*′ = 2). For both mol­ecules, the two amide groups are not coplanar, as the dihedral angles of the respective NCO groups are similar at 50.37 (14) and 51.22 (13)°. However, the orientations of the substituent phenyl rings with the central naphthalene system are significantly different for the two mol­ecules; for one mol­ecule, these dihedral angles are 80.29 (3) and 80.95 (4)°, while for the second mol­ecule they are 86.63 (3) and 72.82 (4)°. The crystal packing shows the mol­ecules to be linked by weak C—H⋯O inter­actions.

## Related literature

For related structures, see: Akinboye, Butcher, Brandy *et al.* (2009 ▶); Akinboye, Butcher, Wright *et al.* (2009 ▶). For pharmacological properties of related compounds, see: Bakare *et al.* (2003 ▶); Khraiwesh *et al.* (2011 ▶); Berhe *et al.* (2008 ▶).
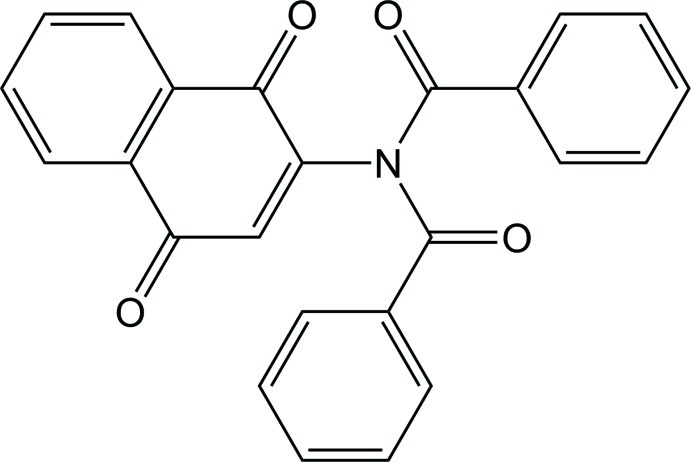


## Experimental

### 

#### Crystal data

C_24_H_15_NO_4_
*M* *_r_* = 381.37
Triclinic, 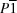

*a* = 9.8704 (11) Å
*b* = 12.6776 (11) Å
*c* = 15.6472 (14) Å
α = 90.735 (7)°
β = 98.213 (9)°
γ = 108.187 (9)°
*V* = 1837.6 (3) Å^3^
*Z* = 4
Mo *K*α radiation
μ = 0.10 mm^−1^
*T* = 123 K
0.52 × 0.28 × 0.15 mm

#### Data collection

Oxford Diffraction Xcalibur Ruby Gemini diffractometer
Absorption correction: multi-scan (*CrysAlis PRO*; Oxford Diffraction, 2007 ▶) *T*_min_ = 0.751, *T*_max_ = 1.000
26547 measured reflections
26547 independent reflections
17970 reflections with *I* > 2σ(*I*)

#### Refinement

*R*[*F* ^2^ > 2σ(*F* ^2^)] = 0.070
*wR*(*F* ^2^) = 0.221
*S* = 1.03
26547 reflections
524 parameters
H-atom parameters constrained
Δρ_max_ = 0.70 e Å^−3^
Δρ_min_ = −0.43 e Å^−3^

### 

Data collection: *CrysAlis PRO* (Oxford Diffraction, 2007 ▶); cell refinement: *CrysAlis PRO*; data reduction: *CrysAlis PRO*; program(s) used to solve structure: *SHELXS97* (Sheldrick, 2008 ▶); program(s) used to refine structure: *SHELXL97* (Sheldrick, 2008 ▶); molecular graphics: *SHELXTL* (Sheldrick, 2008 ▶); software used to prepare material for publication: *SHELXTL*.

## Supplementary Material

Crystal structure: contains datablock(s) I, global. DOI: 10.1107/S1600536812030231/jj2146sup1.cif

Structure factors: contains datablock(s) I. DOI: 10.1107/S1600536812030231/jj2146Isup2.hkl

Supplementary material file. DOI: 10.1107/S1600536812030231/jj2146Isup3.cml

Additional supplementary materials:  crystallographic information; 3D view; checkCIF report

## Figures and Tables

**Table 1:** Hydrogen-bond geometry (Å, °)

*D*—H⋯*A*	*D*—H	H⋯*A*	*D*⋯*A*	*D*—H⋯*A*
C21*A*—H21*A*⋯O2*B* ^i^	0.95	2.41	3.1270 (19)	132
C14*B*—H14*B*⋯O3*A* ^ii^	0.95	2.50	3.4345 (17)	168
C15*B*—H15*B*⋯O4*A* ^iii^	0.95	2.62	3.4638 (17)	148
C22*B*—H22*B*⋯O2*A*	0.95	2.54	3.2054 (17)	127
C14*A*—H14*A*⋯O3*B*	0.95	2.44	3.2303 (16)	140

Symmetry codes: (i) 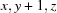; (ii) 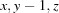; (iii) 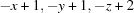.

**Fig. 1.**
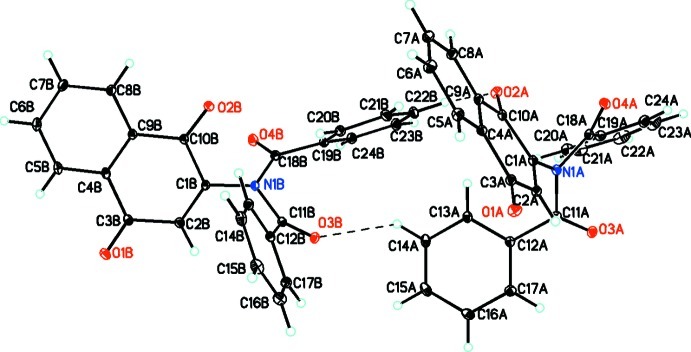
Diagram of C24H15NO4 showing the atom labeling for the two molecules in the asymmetric unit. The C—H···O interactions are shown by dashed lines. Thermal ellipsoids are at the 30% probability level.

**Fig. 2.**
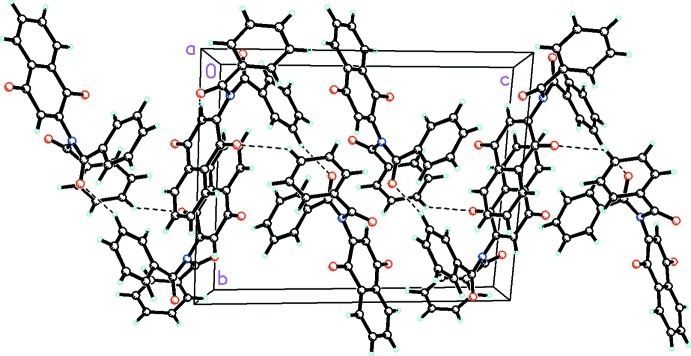
The molecular packing for C24H15NO4 viewed along the a axis. The C—H···O interactions are shown by dashed lines.

## supplementary crystallographic information

### Comment

We have previously synthesized some cyclic and acyclic imido-3-chloro-naphthoquinone analogs and studied their anticancer activities against some prostate cancer cell lines (Bakare *et al.* (2003); Berhe *et al.* (2008)). In addition, some aryl imido -2-chloro and -2-bromo-1,4-naphthoquinone analogs were synthesized and some crystal structures were already reported for the 2-*N*-bis(2-chlorobenzoyl)amino and 2-*N*-bis(4-fluorobenzoyl) amino-3-bromo-1,4-naphthoquinone derivatives (Akinboye, Butcher, Brandy *et al.* (2009); Akinboye, Butcher, Wright *et al.* (2009)). Recently, we have reported some antiparasitic studies of some of the aryl imidonaphthoquinones (Khraiwesh, *et al.*, (2011)) and are currently studying their anticancer properties against PC3 prostate cancer cells. In continuation of our work, *N*-benzoyl-*N*-(1,4-dioxonaphthalen-2-yl)benzamide was synthesized as a potential antiprostate cancer agent and its structure is reported here.

In the structure of the title compound there are two molecules in the asymmetric unit (*Z*' = 2). For both molecules the two amide moieties are not coplanar as the dihedral angles of the respective NCO groups are similar at 50.37 (14)° and 51.22 (13)°). However, the orientations of the substituent phenyl rings with the central naphthyl ring are significantly different for the two molecules. For molecule A these dihedral angles are 80.29 (3)° and 80.95 (4)° between the naphthyl ring and rings C12 - C17 and C19 - C24, while for molecule B these values are 86.63 (3)° and 72.82 (4)°.

In the two molecules the imide C=O's are at dihedral angles of 42.95 (17)° and -121.12 (13)° for molecule A and -44.89 (17)° and 121.05 (13)° for molecule B and are thus anti to each other. The anti conformation of these imide C=O's and the dihedral angles between the phenyl rings and imide C=O groups oriented the phenyl groups away from each other (values of (34.2 (2)° and 25.29 (18)°) for A and -24.03 (17) and -42.44 (19)° for B). The crystal structure packing pattern shows the molecules were linked by weak intermolecular C—H···O interactions.

### Experimental

2-Amino-1,4-naphthoquinone (318 mg, 1.83 mmol) was dissolved in freshly distilled THF (15 ml). NaH (115 mg, 4.78 mmol) was added and the mixture was stirred at room temperature for 15 min. The appropriate benzoyl chloride (0.55 ml, 4.74 mmol) was added, drop wise, and the mixture was stirred for 24 h. THF was evaporated under vacuum and the mixture was washed with ice-water (10 g ice in 10 ml water). The aqueous mixture was extracted with CH_2_Cl_2_ (30 ml, 20 ml consecutively) and the combined organic phase washed with water (3 *x* 20 ml), saturated NaCl solution (20 ml), then dried over anhydrous MgSO_4_. The crude was purified *via* tirturating in ethanol (2 ml) and column chromatography with an eluent mixture of ethyl acetate and hexane to furnish the imide(70 mg, 10%).

### Refinement

H atoms were placed in geometrically idealized positions and constrained to ride on their parent atoms with a C—H distance of 0.95 and *U*_iso_(H) = 1.2*U*_eq_(C). The structure was a non-merohedral twin and was refined with a BASF (batch scale factor) value of 0.4084 (7) and a twin law for the two components of -1 0 0, 0 - 1 0, 0 0 1.

### Crystal data

**Table:**

C_24_H_15_NO_4_	*Z* = 4
*M**_r_* = 381.37	*F*(000) = 792
Triclinic, *P*1	*D*_x_ = 1.378 Mg m^−^^3^
Hall symbol: -P 1	Mo *K*α radiation, λ = 0.71073 Å
*a* = 9.8704 (11) Å	Cell parameters from 3142 reflections
*b* = 12.6776 (11) Å	θ = 3.0–34.9°
*c* = 15.6472 (14) Å	µ = 0.10 mm^−^^1^
α = 90.735 (7)°	*T* = 123 K
β = 98.213 (9)°	Block, colorless
γ = 108.187 (9)°	0.52 × 0.28 × 0.15 mm
*V* = 1837.6 (3) Å^3^	

### Data collection

**Table:**

Oxford Diffraction Xcalibur Ruby Gemini diffractometer	26547 independent reflections
Radiation source: Enhance (Mo) X-ray Source	17970 reflections with *I* > 2σ(*I*)
Graphite monochromator	*R*_int_ = 0.000
Detector resolution: 10.5081 pixels mm^-1^	θ_max_ = 35.2°, θ_min_ = 3.1°
ω scans	*h* = −15→15
Absorption correction: multi-scan (*CrysAlis PRO*; Oxford Diffraction, 2007)	*k* = −20→20
*T*_min_ = 0.751, *T*_max_ = 1.000	*l* = −24→21
26547 measured reflections	

### Refinement

**Table:**

Refinement on *F*^2^	Primary atom site location: structure-invariant direct methods
Least-squares matrix: full	Secondary atom site location: difference Fourier map
*R*[*F*^2^ > 2σ(*F*^2^)] = 0.070	Hydrogen site location: inferred from neighbouring sites
*wR*(*F*^2^) = 0.221	H-atom parameters constrained
*S* = 1.03	*w* = 1/[σ^2^(*F*_o_^2^) + (0.1412*P*)^2^] where *P* = (*F*_o_^2^ + 2*F*_c_^2^)/3
26547 reflections	(Δ/σ)_max_ = 0.001
524 parameters	Δρ_max_ = 0.70 e Å^−^^3^
0 restraints	Δρ_min_ = −0.43 e Å^−^^3^

### Special details

**Table:**

Geometry. All e.s.d.'s (except the e.s.d. in the dihedral angle between two l.s. planes) are estimated using the full covariance matrix. The cell e.s.d.'s are taken into account individually in the estimation of e.s.d.'s in distances, angles and torsion angles; correlations between e.s.d.'s in cell parameters are only used when they are defined by crystal symmetry. An approximate (isotropic) treatment of cell e.s.d.'s is used for estimating e.s.d.'s involving l.s. planes.
Refinement. Refinement of *F*^2^ against ALL reflections. The weighted *R*-factor *wR* and goodness of fit *S* are based on *F*^2^, conventional *R*-factors *R* are based on *F*, with *F* set to zero for negative *F*^2^. The threshold expression of *F*^2^ > σ(*F*^2^) is used only for calculating *R*-factors(gt) *etc*. and is not relevant to the choice of reflections for refinement. *R*-factors based on *F*^2^ are statistically about twice as large as those based on *F*, and *R*- factors based on ALL data will be even larger.

### Fractional atomic coordinates and isotropic or equivalent isotropic displacement parameters (Å^2^)

**Table:**

	*x*	*y*	*z*	*U*_iso_*/*U*_eq_	
O1A	0.61934 (11)	0.65075 (8)	1.11669 (6)	0.0244 (2)	
O2A	0.15230 (10)	0.64038 (8)	0.89442 (6)	0.02104 (19)	
O3A	0.50651 (10)	1.01393 (8)	0.90297 (6)	0.02088 (19)	
O4A	0.18605 (11)	0.84042 (8)	1.00964 (6)	0.02152 (19)	
N1A	0.36272 (12)	0.84143 (9)	0.93028 (7)	0.0166 (2)	
C1A	0.37834 (13)	0.74566 (10)	0.97147 (7)	0.0153 (2)	
C2A	0.49758 (14)	0.74697 (10)	1.02443 (8)	0.0173 (2)	
H2AA	0.5767	0.8139	1.0342	0.021*	
C3A	0.50990 (14)	0.64715 (10)	1.06827 (8)	0.0173 (2)	
C4A	0.38274 (13)	0.54455 (10)	1.05355 (7)	0.0160 (2)	
C5A	0.38512 (15)	0.45054 (11)	1.09716 (9)	0.0209 (2)	
H5AA	0.4686	0.4515	1.1362	0.025*	
C6A	0.26582 (16)	0.35463 (11)	1.08401 (9)	0.0240 (3)	
H6AA	0.2685	0.2902	1.1137	0.029*	
C7A	0.14371 (16)	0.35298 (11)	1.02791 (9)	0.0230 (3)	
H7AA	0.0620	0.2877	1.0195	0.028*	
C8A	0.14043 (15)	0.44648 (11)	0.98398 (8)	0.0198 (2)	
H8AA	0.0567	0.4450	0.9449	0.024*	
C9A	0.25916 (14)	0.54244 (10)	0.99677 (7)	0.0158 (2)	
C10A	0.25331 (13)	0.64191 (10)	0.94990 (7)	0.0155 (2)	
C11A	0.47714 (14)	0.91402 (10)	0.89268 (7)	0.0165 (2)	
C12A	0.55113 (14)	0.86068 (10)	0.83735 (8)	0.0166 (2)	
C13A	0.47877 (15)	0.75844 (11)	0.79278 (8)	0.0187 (2)	
H13A	0.3835	0.7184	0.8017	0.022*	
C14A	0.54511 (16)	0.71467 (12)	0.73529 (8)	0.0226 (3)	
H14A	0.4952	0.6450	0.7046	0.027*	
C15A	0.68457 (17)	0.77295 (13)	0.72280 (8)	0.0251 (3)	
H15A	0.7307	0.7429	0.6840	0.030*	
C16A	0.75694 (16)	0.87551 (13)	0.76714 (9)	0.0253 (3)	
H16A	0.8525	0.9152	0.7585	0.030*	
C17A	0.69028 (15)	0.92022 (11)	0.82395 (8)	0.0207 (2)	
H17A	0.7392	0.9908	0.8534	0.025*	
C18A	0.24559 (14)	0.87524 (10)	0.94867 (8)	0.0169 (2)	
C19A	0.19812 (14)	0.95011 (11)	0.88779 (9)	0.0206 (2)	
C20A	0.20504 (17)	0.94166 (13)	0.79946 (9)	0.0271 (3)	
H20A	0.2413	0.8876	0.7771	0.033*	
C21A	0.15885 (19)	1.01249 (15)	0.74495 (11)	0.0367 (4)	
H21A	0.1623	1.0066	0.6848	0.044*	
C22A	0.10775 (19)	1.09162 (14)	0.77739 (13)	0.0410 (4)	
H22A	0.0791	1.1415	0.7397	0.049*	
C23A	0.09770 (19)	1.09917 (14)	0.86423 (13)	0.0389 (4)	
H23A	0.0609	1.1532	0.8860	0.047*	
C24A	0.14178 (16)	1.02722 (12)	0.91974 (10)	0.0268 (3)	
H24A	0.1333	1.0309	0.9793	0.032*	
O1B	0.60121 (11)	0.13107 (9)	0.39332 (6)	0.0239 (2)	
O2B	0.18809 (11)	0.14122 (8)	0.57590 (6)	0.02112 (19)	
O3B	0.53144 (11)	0.51594 (8)	0.59971 (6)	0.01961 (18)	
O4B	0.18878 (11)	0.32712 (8)	0.45919 (6)	0.0232 (2)	
N1B	0.38724 (11)	0.33871 (9)	0.55734 (6)	0.01598 (19)	
C1B	0.39502 (13)	0.23977 (10)	0.51787 (7)	0.0149 (2)	
C2B	0.50258 (14)	0.23682 (11)	0.47547 (8)	0.0181 (2)	
H2BA	0.5809	0.3029	0.4738	0.022*	
C3B	0.50210 (14)	0.13280 (11)	0.43110 (8)	0.0176 (2)	
C4B	0.37397 (14)	0.03230 (10)	0.43147 (7)	0.0170 (2)	
C5B	0.36221 (16)	−0.06502 (11)	0.38440 (8)	0.0216 (3)	
H5BA	0.4353	−0.0670	0.3515	0.026*	
C6B	0.24392 (16)	−0.15902 (12)	0.38559 (9)	0.0248 (3)	
H6BA	0.2356	−0.2249	0.3528	0.030*	
C7B	0.13726 (15)	−0.15763 (11)	0.43448 (9)	0.0242 (3)	
H7BA	0.0570	−0.2226	0.4354	0.029*	
C8B	0.14839 (15)	−0.06103 (11)	0.48195 (9)	0.0215 (2)	
H8BA	0.0764	−0.0600	0.5160	0.026*	
C9B	0.26546 (13)	0.03420 (10)	0.47945 (8)	0.0163 (2)	
C10B	0.27458 (13)	0.13758 (10)	0.52801 (7)	0.0157 (2)	
C11B	0.50716 (13)	0.41715 (10)	0.60839 (7)	0.0155 (2)	
C12B	0.59360 (13)	0.37346 (10)	0.67570 (7)	0.0164 (2)	
C13B	0.53263 (14)	0.27461 (11)	0.71402 (8)	0.0179 (2)	
H13B	0.4363	0.2297	0.6932	0.021*	
C14B	0.61293 (16)	0.24183 (12)	0.78280 (8)	0.0226 (3)	
H14B	0.5723	0.1742	0.8088	0.027*	
C15B	0.75295 (17)	0.30876 (13)	0.81313 (8)	0.0258 (3)	
H15B	0.8081	0.2866	0.8601	0.031*	
C16B	0.81343 (16)	0.40773 (13)	0.77565 (8)	0.0247 (3)	
H16B	0.9095	0.4529	0.7968	0.030*	
C17B	0.73313 (14)	0.44047 (11)	0.70715 (8)	0.0199 (2)	
H17B	0.7736	0.5087	0.6818	0.024*	
C18B	0.26274 (14)	0.36802 (10)	0.52716 (8)	0.0174 (2)	
C19B	0.22434 (13)	0.44303 (10)	0.58654 (8)	0.0176 (2)	
C20B	0.23316 (15)	0.42713 (11)	0.67508 (8)	0.0211 (2)	
H20B	0.2701	0.3713	0.6990	0.025*	
C21B	0.18735 (16)	0.49364 (13)	0.72766 (9)	0.0251 (3)	
H21B	0.1918	0.4826	0.7878	0.030*	
C22B	0.13520 (16)	0.57591 (13)	0.69353 (10)	0.0266 (3)	
H22B	0.1049	0.6215	0.7303	0.032*	
C23B	0.12693 (16)	0.59214 (12)	0.60551 (10)	0.0257 (3)	
H23B	0.0923	0.6493	0.5821	0.031*	
C24B	0.16968 (15)	0.52406 (11)	0.55198 (9)	0.0215 (3)	
H24B	0.1613	0.5332	0.4915	0.026*	

### Atomic displacement parameters (Å^2^)

**Table:**

	*U*^11^	*U*^22^	*U*^33^	*U*^12^	*U*^13^	*U*^23^
O1A	0.0214 (5)	0.0244 (5)	0.0260 (5)	0.0075 (4)	−0.0014 (4)	0.0038 (4)
O2A	0.0221 (5)	0.0188 (4)	0.0199 (4)	0.0053 (4)	−0.0014 (3)	0.0022 (3)
O3A	0.0260 (5)	0.0144 (4)	0.0220 (4)	0.0050 (4)	0.0060 (4)	0.0017 (3)
O4A	0.0227 (5)	0.0232 (5)	0.0195 (4)	0.0068 (4)	0.0074 (3)	0.0027 (4)
N1A	0.0217 (5)	0.0134 (4)	0.0162 (4)	0.0065 (4)	0.0057 (4)	0.0035 (4)
C1A	0.0192 (6)	0.0133 (5)	0.0144 (5)	0.0052 (4)	0.0049 (4)	0.0027 (4)
C2A	0.0181 (6)	0.0146 (5)	0.0184 (5)	0.0036 (4)	0.0038 (4)	0.0004 (4)
C3A	0.0185 (6)	0.0174 (5)	0.0163 (5)	0.0056 (4)	0.0034 (4)	0.0007 (4)
C4A	0.0178 (5)	0.0156 (5)	0.0154 (5)	0.0060 (4)	0.0041 (4)	0.0022 (4)
C5A	0.0237 (6)	0.0189 (6)	0.0217 (6)	0.0089 (5)	0.0033 (5)	0.0047 (5)
C6A	0.0289 (7)	0.0163 (6)	0.0272 (6)	0.0067 (5)	0.0062 (5)	0.0085 (5)
C7A	0.0245 (7)	0.0149 (5)	0.0287 (7)	0.0036 (5)	0.0074 (5)	0.0034 (5)
C8A	0.0197 (6)	0.0171 (5)	0.0219 (6)	0.0054 (5)	0.0025 (4)	0.0007 (4)
C9A	0.0191 (6)	0.0141 (5)	0.0151 (5)	0.0060 (4)	0.0040 (4)	0.0011 (4)
C10A	0.0193 (6)	0.0139 (5)	0.0140 (5)	0.0060 (4)	0.0035 (4)	0.0007 (4)
C11A	0.0196 (6)	0.0161 (5)	0.0134 (5)	0.0052 (4)	0.0027 (4)	0.0022 (4)
C12A	0.0211 (6)	0.0157 (5)	0.0142 (5)	0.0069 (4)	0.0042 (4)	0.0027 (4)
C13A	0.0243 (6)	0.0175 (5)	0.0153 (5)	0.0073 (5)	0.0044 (4)	0.0017 (4)
C14A	0.0323 (7)	0.0227 (6)	0.0156 (5)	0.0127 (5)	0.0037 (5)	−0.0005 (5)
C15A	0.0330 (8)	0.0327 (7)	0.0180 (6)	0.0197 (6)	0.0094 (5)	0.0056 (5)
C16A	0.0239 (7)	0.0294 (7)	0.0258 (6)	0.0100 (6)	0.0106 (5)	0.0083 (5)
C17A	0.0222 (6)	0.0197 (6)	0.0205 (6)	0.0054 (5)	0.0064 (5)	0.0051 (5)
C18A	0.0198 (6)	0.0138 (5)	0.0170 (5)	0.0054 (4)	0.0025 (4)	0.0001 (4)
C19A	0.0194 (6)	0.0186 (6)	0.0239 (6)	0.0067 (5)	0.0023 (5)	0.0040 (5)
C20A	0.0285 (7)	0.0295 (7)	0.0232 (6)	0.0092 (6)	0.0032 (5)	0.0074 (5)
C21A	0.0333 (9)	0.0418 (9)	0.0315 (8)	0.0086 (7)	0.0001 (6)	0.0172 (7)
C22A	0.0337 (9)	0.0283 (8)	0.0546 (11)	0.0075 (7)	−0.0095 (8)	0.0182 (8)
C23A	0.0348 (9)	0.0237 (7)	0.0571 (11)	0.0149 (7)	−0.0085 (8)	0.0021 (7)
C24A	0.0244 (7)	0.0212 (6)	0.0355 (7)	0.0105 (5)	−0.0001 (6)	−0.0019 (6)
O1B	0.0253 (5)	0.0267 (5)	0.0251 (5)	0.0130 (4)	0.0103 (4)	0.0033 (4)
O2B	0.0216 (5)	0.0201 (4)	0.0223 (4)	0.0056 (4)	0.0079 (4)	−0.0004 (4)
O3B	0.0238 (5)	0.0150 (4)	0.0200 (4)	0.0059 (3)	0.0039 (3)	0.0006 (3)
O4B	0.0240 (5)	0.0264 (5)	0.0197 (4)	0.0112 (4)	−0.0021 (4)	−0.0034 (4)
N1B	0.0171 (5)	0.0149 (5)	0.0164 (4)	0.0062 (4)	0.0018 (4)	−0.0007 (4)
C1B	0.0176 (5)	0.0134 (5)	0.0146 (5)	0.0066 (4)	0.0015 (4)	0.0004 (4)
C2B	0.0198 (6)	0.0179 (5)	0.0178 (5)	0.0070 (5)	0.0047 (4)	0.0020 (4)
C3B	0.0201 (6)	0.0198 (6)	0.0161 (5)	0.0104 (5)	0.0038 (4)	0.0029 (4)
C4B	0.0213 (6)	0.0180 (5)	0.0144 (5)	0.0105 (5)	0.0012 (4)	0.0011 (4)
C5B	0.0271 (7)	0.0208 (6)	0.0199 (6)	0.0125 (5)	0.0025 (5)	−0.0024 (5)
C6B	0.0307 (7)	0.0189 (6)	0.0258 (6)	0.0116 (5)	−0.0004 (5)	−0.0050 (5)
C7B	0.0218 (6)	0.0157 (6)	0.0317 (7)	0.0040 (5)	−0.0021 (5)	−0.0029 (5)
C8B	0.0191 (6)	0.0183 (6)	0.0269 (6)	0.0066 (5)	0.0017 (5)	−0.0015 (5)
C9B	0.0177 (6)	0.0153 (5)	0.0169 (5)	0.0073 (4)	0.0007 (4)	−0.0002 (4)
C10B	0.0166 (5)	0.0154 (5)	0.0157 (5)	0.0061 (4)	0.0017 (4)	0.0004 (4)
C11B	0.0172 (5)	0.0167 (5)	0.0137 (5)	0.0063 (4)	0.0035 (4)	0.0001 (4)
C12B	0.0183 (6)	0.0184 (5)	0.0137 (5)	0.0073 (4)	0.0029 (4)	−0.0001 (4)
C13B	0.0212 (6)	0.0170 (5)	0.0157 (5)	0.0057 (4)	0.0042 (4)	0.0007 (4)
C14B	0.0344 (7)	0.0233 (6)	0.0149 (5)	0.0148 (6)	0.0066 (5)	0.0034 (5)
C15B	0.0326 (8)	0.0350 (8)	0.0153 (5)	0.0194 (6)	0.0017 (5)	0.0035 (5)
C16B	0.0233 (7)	0.0324 (7)	0.0184 (6)	0.0104 (6)	0.0000 (5)	0.0013 (5)
C17B	0.0203 (6)	0.0216 (6)	0.0171 (5)	0.0063 (5)	0.0018 (4)	0.0000 (4)
C18B	0.0172 (6)	0.0171 (5)	0.0192 (5)	0.0073 (4)	0.0025 (4)	0.0017 (4)
C19B	0.0159 (5)	0.0179 (5)	0.0200 (5)	0.0065 (4)	0.0029 (4)	−0.0016 (4)
C20B	0.0207 (6)	0.0225 (6)	0.0212 (6)	0.0079 (5)	0.0047 (5)	0.0007 (5)
C21B	0.0239 (7)	0.0299 (7)	0.0228 (6)	0.0088 (6)	0.0071 (5)	−0.0020 (5)
C22B	0.0228 (7)	0.0274 (7)	0.0308 (7)	0.0103 (5)	0.0045 (5)	−0.0098 (6)
C23B	0.0243 (7)	0.0239 (7)	0.0314 (7)	0.0134 (5)	−0.0001 (5)	−0.0030 (5)
C24B	0.0198 (6)	0.0228 (6)	0.0229 (6)	0.0097 (5)	−0.0001 (5)	−0.0003 (5)

### Geometric parameters (Å, º)

**Table:**

O1A—C3A	1.2154 (16)	O1B—C3B	1.2188 (15)
O2A—C10A	1.2199 (15)	O2B—C10B	1.2243 (15)
O3A—C11A	1.2109 (15)	O3B—C11B	1.2127 (15)
O4A—C18A	1.2080 (15)	O4B—C18B	1.2086 (15)
N1A—C18A	1.4158 (16)	N1B—C18B	1.4125 (16)
N1A—C1A	1.4218 (15)	N1B—C11B	1.4152 (16)
N1A—C11A	1.4226 (16)	N1B—C1B	1.4192 (15)
C1A—C2A	1.3345 (17)	C1B—C2B	1.3395 (17)
C1A—C10A	1.4911 (18)	C1B—C10B	1.4882 (17)
C2A—C3A	1.4769 (18)	C2B—C3B	1.4807 (17)
C2A—H2AA	0.9500	C2B—H2BA	0.9500
C3A—C4A	1.4869 (18)	C3B—C4B	1.4897 (19)
C4A—C5A	1.3858 (17)	C4B—C5B	1.3933 (17)
C4A—C9A	1.3955 (17)	C4B—C9B	1.3988 (17)
C5A—C6A	1.392 (2)	C5B—C6B	1.386 (2)
C5A—H5AA	0.9500	C5B—H5BA	0.9500
C6A—C7A	1.380 (2)	C6B—C7B	1.391 (2)
C6A—H6AA	0.9500	C6B—H6BA	0.9500
C7A—C8A	1.3846 (18)	C7B—C8B	1.3903 (18)
C7A—H7AA	0.9500	C7B—H7BA	0.9500
C8A—C9A	1.3893 (18)	C8B—C9B	1.3920 (18)
C8A—H8AA	0.9500	C8B—H8BA	0.9500
C9A—C10A	1.4793 (17)	C9B—C10B	1.4780 (16)
C11A—C12A	1.4880 (17)	C11B—C12B	1.4824 (17)
C12A—C13A	1.3904 (18)	C12B—C17B	1.3877 (18)
C12A—C17A	1.3926 (18)	C12B—C13B	1.3942 (18)
C13A—C14A	1.3886 (18)	C13B—C14B	1.3901 (18)
C13A—H13A	0.9500	C13B—H13B	0.9500
C14A—C15A	1.387 (2)	C14B—C15B	1.388 (2)
C14A—H14A	0.9500	C14B—H14B	0.9500
C15A—C16A	1.392 (2)	C15B—C16B	1.388 (2)
C15A—H15A	0.9500	C15B—H15B	0.9500
C16A—C17A	1.3897 (18)	C16B—C17B	1.3869 (18)
C16A—H16A	0.9500	C16B—H16B	0.9500
C17A—H17A	0.9500	C17B—H17B	0.9500
C18A—C19A	1.4836 (18)	C18B—C19B	1.4881 (17)
C19A—C24A	1.3860 (19)	C19B—C24B	1.3841 (18)
C19A—C20A	1.398 (2)	C19B—C20B	1.3967 (18)
C20A—C21A	1.380 (2)	C20B—C21B	1.3852 (18)
C20A—H20A	0.9500	C20B—H20B	0.9500
C21A—C22A	1.376 (3)	C21B—C22B	1.382 (2)
C21A—H21A	0.9500	C21B—H21B	0.9500
C22A—C23A	1.381 (3)	C22B—C23B	1.390 (2)
C22A—H22A	0.9500	C22B—H22B	0.9500
C23A—C24A	1.391 (2)	C23B—C24B	1.3907 (19)
C23A—H23A	0.9500	C23B—H23B	0.9500
C24A—H24A	0.9500	C24B—H24B	0.9500
			
C18A—N1A—C1A	115.64 (10)	C18B—N1B—C11B	119.88 (10)
C18A—N1A—C11A	121.20 (10)	C18B—N1B—C1B	115.62 (10)
C1A—N1A—C11A	121.16 (11)	C11B—N1B—C1B	123.00 (10)
C2A—C1A—N1A	122.76 (11)	C2B—C1B—N1B	123.34 (11)
C2A—C1A—C10A	121.76 (11)	C2B—C1B—C10B	121.83 (11)
N1A—C1A—C10A	115.46 (11)	N1B—C1B—C10B	114.83 (10)
C1A—C2A—C3A	121.80 (11)	C1B—C2B—C3B	121.46 (12)
C1A—C2A—H2AA	119.1	C1B—C2B—H2BA	119.3
C3A—C2A—H2AA	119.1	C3B—C2B—H2BA	119.3
O1A—C3A—C2A	120.50 (12)	O1B—C3B—C2B	120.78 (12)
O1A—C3A—C4A	121.68 (12)	O1B—C3B—C4B	121.54 (12)
C2A—C3A—C4A	117.79 (11)	C2B—C3B—C4B	117.63 (11)
C5A—C4A—C9A	119.36 (12)	C5B—C4B—C9B	119.45 (12)
C5A—C4A—C3A	120.22 (11)	C5B—C4B—C3B	119.95 (12)
C9A—C4A—C3A	120.41 (11)	C9B—C4B—C3B	120.60 (11)
C4A—C5A—C6A	120.35 (13)	C6B—C5B—C4B	120.00 (13)
C4A—C5A—H5AA	119.8	C6B—C5B—H5BA	120.0
C6A—C5A—H5AA	119.8	C4B—C5B—H5BA	120.0
C7A—C6A—C5A	120.09 (12)	C5B—C6B—C7B	120.52 (12)
C7A—C6A—H6AA	120.0	C5B—C6B—H6BA	119.7
C5A—C6A—H6AA	120.0	C7B—C6B—H6BA	119.7
C6A—C7A—C8A	119.95 (13)	C8B—C7B—C6B	119.88 (13)
C6A—C7A—H7AA	120.0	C8B—C7B—H7BA	120.1
C8A—C7A—H7AA	120.0	C6B—C7B—H7BA	120.1
C7A—C8A—C9A	120.24 (13)	C7B—C8B—C9B	119.75 (13)
C7A—C8A—H8AA	119.9	C7B—C8B—H8BA	120.1
C9A—C8A—H8AA	119.9	C9B—C8B—H8BA	120.1
C8A—C9A—C4A	120.00 (11)	C8B—C9B—C4B	120.37 (11)
C8A—C9A—C10A	119.53 (11)	C8B—C9B—C10B	119.53 (11)
C4A—C9A—C10A	120.48 (11)	C4B—C9B—C10B	120.10 (11)
O2A—C10A—C9A	122.29 (11)	O2B—C10B—C9B	122.45 (11)
O2A—C10A—C1A	120.24 (11)	O2B—C10B—C1B	119.78 (11)
C9A—C10A—C1A	117.45 (11)	C9B—C10B—C1B	117.77 (11)
O3A—C11A—N1A	120.52 (11)	O3B—C11B—N1B	120.39 (11)
O3A—C11A—C12A	122.84 (12)	O3B—C11B—C12B	122.35 (11)
N1A—C11A—C12A	116.56 (11)	N1B—C11B—C12B	117.14 (11)
C13A—C12A—C17A	120.08 (12)	C17B—C12B—C13B	120.25 (12)
C13A—C12A—C11A	121.06 (12)	C17B—C12B—C11B	117.80 (12)
C17A—C12A—C11A	118.55 (12)	C13B—C12B—C11B	121.52 (11)
C14A—C13A—C12A	120.29 (13)	C14B—C13B—C12B	119.90 (13)
C14A—C13A—H13A	119.9	C14B—C13B—H13B	120.1
C12A—C13A—H13A	119.9	C12B—C13B—H13B	120.1
C15A—C14A—C13A	119.78 (13)	C15B—C14B—C13B	119.42 (13)
C15A—C14A—H14A	120.1	C15B—C14B—H14B	120.3
C13A—C14A—H14A	120.1	C13B—C14B—H14B	120.3
C14A—C15A—C16A	119.99 (12)	C14B—C15B—C16B	120.80 (13)
C14A—C15A—H15A	120.0	C14B—C15B—H15B	119.6
C16A—C15A—H15A	120.0	C16B—C15B—H15B	119.6
C17A—C16A—C15A	120.40 (13)	C17B—C16B—C15B	119.74 (14)
C17A—C16A—H16A	119.8	C17B—C16B—H16B	120.1
C15A—C16A—H16A	119.8	C15B—C16B—H16B	120.1
C16A—C17A—C12A	119.45 (13)	C16B—C17B—C12B	119.88 (13)
C16A—C17A—H17A	120.3	C16B—C17B—H17B	120.1
C12A—C17A—H17A	120.3	C12B—C17B—H17B	120.1
O4A—C18A—N1A	120.27 (11)	O4B—C18B—N1B	120.19 (11)
O4A—C18A—C19A	123.00 (12)	O4B—C18B—C19B	123.43 (12)
N1A—C18A—C19A	116.68 (11)	N1B—C18B—C19B	116.20 (11)
C24A—C19A—C20A	120.03 (13)	C24B—C19B—C20B	120.16 (12)
C24A—C19A—C18A	118.57 (12)	C24B—C19B—C18B	118.96 (11)
C20A—C19A—C18A	121.36 (12)	C20B—C19B—C18B	120.72 (11)
C21A—C20A—C19A	119.50 (15)	C21B—C20B—C19B	119.25 (13)
C21A—C20A—H20A	120.3	C21B—C20B—H20B	120.4
C19A—C20A—H20A	120.3	C19B—C20B—H20B	120.4
C22A—C21A—C20A	120.30 (16)	C22B—C21B—C20B	120.66 (13)
C22A—C21A—H21A	119.8	C22B—C21B—H21B	119.7
C20A—C21A—H21A	119.8	C20B—C21B—H21B	119.7
C21A—C22A—C23A	120.65 (15)	C21B—C22B—C23B	120.15 (13)
C21A—C22A—H22A	119.7	C21B—C22B—H22B	119.9
C23A—C22A—H22A	119.7	C23B—C22B—H22B	119.9
C22A—C23A—C24A	119.68 (16)	C22B—C23B—C24B	119.52 (13)
C22A—C23A—H23A	120.2	C22B—C23B—H23B	120.2
C24A—C23A—H23A	120.2	C24B—C23B—H23B	120.2
C19A—C24A—C23A	119.77 (15)	C19B—C24B—C23B	120.22 (13)
C19A—C24A—H24A	120.1	C19B—C24B—H24B	119.9
C23A—C24A—H24A	120.1	C23B—C24B—H24B	119.9
			
C18A—N1A—C1A—C2A	−121.12 (13)	C18B—N1B—C1B—C2B	121.05 (13)
C11A—N1A—C1A—C2A	42.95 (17)	C11B—N1B—C1B—C2B	−44.89 (17)
C18A—N1A—C1A—C10A	60.33 (14)	C18B—N1B—C1B—C10B	−59.77 (14)
C11A—N1A—C1A—C10A	−135.60 (11)	C11B—N1B—C1B—C10B	134.29 (12)
N1A—C1A—C2A—C3A	178.38 (11)	N1B—C1B—C2B—C3B	−176.55 (11)
C10A—C1A—C2A—C3A	−3.15 (18)	C10B—C1B—C2B—C3B	4.32 (18)
C1A—C2A—C3A—O1A	−179.48 (12)	C1B—C2B—C3B—O1B	−179.43 (12)
C1A—C2A—C3A—C4A	−1.66 (18)	C1B—C2B—C3B—C4B	2.96 (17)
O1A—C3A—C4A—C5A	1.75 (19)	O1B—C3B—C4B—C5B	−3.17 (18)
C2A—C3A—C4A—C5A	−176.04 (11)	C2B—C3B—C4B—C5B	174.43 (11)
O1A—C3A—C4A—C9A	−179.21 (12)	O1B—C3B—C4B—C9B	176.20 (12)
C2A—C3A—C4A—C9A	2.99 (17)	C2B—C3B—C4B—C9B	−6.21 (17)
C9A—C4A—C5A—C6A	0.46 (19)	C9B—C4B—C5B—C6B	−0.20 (19)
C3A—C4A—C5A—C6A	179.50 (12)	C3B—C4B—C5B—C6B	179.17 (12)
C4A—C5A—C6A—C7A	−0.6 (2)	C4B—C5B—C6B—C7B	−0.9 (2)
C5A—C6A—C7A—C8A	0.7 (2)	C5B—C6B—C7B—C8B	0.6 (2)
C6A—C7A—C8A—C9A	−0.6 (2)	C6B—C7B—C8B—C9B	0.8 (2)
C7A—C8A—C9A—C4A	0.51 (19)	C7B—C8B—C9B—C4B	−1.84 (19)
C7A—C8A—C9A—C10A	−179.45 (12)	C7B—C8B—C9B—C10B	178.24 (12)
C5A—C4A—C9A—C8A	−0.42 (18)	C5B—C4B—C9B—C8B	1.57 (18)
C3A—C4A—C9A—C8A	−179.46 (11)	C3B—C4B—C9B—C8B	−177.80 (12)
C5A—C4A—C9A—C10A	179.53 (11)	C5B—C4B—C9B—C10B	−178.51 (11)
C3A—C4A—C9A—C10A	0.49 (17)	C3B—C4B—C9B—C10B	2.11 (17)
C8A—C9A—C10A—O2A	−6.40 (18)	C8B—C9B—C10B—O2B	5.41 (19)
C4A—C9A—C10A—O2A	173.65 (11)	C4B—C9B—C10B—O2B	−174.51 (11)
C8A—C9A—C10A—C1A	174.88 (11)	C8B—C9B—C10B—C1B	−175.12 (11)
C4A—C9A—C10A—C1A	−5.08 (17)	C4B—C9B—C10B—C1B	4.96 (17)
C2A—C1A—C10A—O2A	−172.23 (12)	C2B—C1B—C10B—O2B	171.11 (12)
N1A—C1A—C10A—O2A	6.34 (17)	N1B—C1B—C10B—O2B	−8.09 (16)
C2A—C1A—C10A—C9A	6.52 (17)	C2B—C1B—C10B—C9B	−8.38 (17)
N1A—C1A—C10A—C9A	−174.91 (10)	N1B—C1B—C10B—C9B	172.42 (10)
C18A—N1A—C11A—O3A	26.32 (17)	C18B—N1B—C11B—O3B	−29.07 (17)
C1A—N1A—C11A—O3A	−136.86 (12)	C1B—N1B—C11B—O3B	136.30 (12)
C18A—N1A—C11A—C12A	−150.51 (11)	C18B—N1B—C11B—C12B	146.89 (11)
C1A—N1A—C11A—C12A	46.31 (15)	C1B—N1B—C11B—C12B	−47.74 (15)
O3A—C11A—C12A—C13A	−148.30 (13)	O3B—C11B—C12B—C17B	−24.03 (17)
N1A—C11A—C12A—C13A	28.45 (17)	N1B—C11B—C12B—C17B	160.09 (11)
O3A—C11A—C12A—C17A	25.29 (18)	O3B—C11B—C12B—C13B	148.41 (12)
N1A—C11A—C12A—C17A	−157.96 (11)	N1B—C11B—C12B—C13B	−27.47 (16)
C17A—C12A—C13A—C14A	0.56 (19)	C17B—C12B—C13B—C14B	−1.38 (18)
C11A—C12A—C13A—C14A	174.05 (11)	C11B—C12B—C13B—C14B	−173.64 (11)
C12A—C13A—C14A—C15A	0.46 (19)	C12B—C13B—C14B—C15B	0.62 (19)
C13A—C14A—C15A—C16A	−0.7 (2)	C13B—C14B—C15B—C16B	0.0 (2)
C14A—C15A—C16A—C17A	−0.1 (2)	C14B—C15B—C16B—C17B	0.1 (2)
C15A—C16A—C17A—C12A	1.1 (2)	C15B—C16B—C17B—C12B	−0.9 (2)
C13A—C12A—C17A—C16A	−1.33 (19)	C13B—C12B—C17B—C16B	1.51 (19)
C11A—C12A—C17A—C16A	−174.98 (11)	C11B—C12B—C17B—C16B	174.05 (11)
C1A—N1A—C18A—O4A	16.11 (17)	C11B—N1B—C18B—O4B	149.16 (12)
C11A—N1A—C18A—O4A	−147.96 (12)	C1B—N1B—C18B—O4B	−17.25 (17)
C1A—N1A—C18A—C19A	−161.39 (11)	C11B—N1B—C18B—C19B	−35.53 (16)
C11A—N1A—C18A—C19A	34.55 (16)	C1B—N1B—C18B—C19B	158.06 (11)
O4A—C18A—C19A—C24A	34.2 (2)	O4B—C18B—C19B—C24B	−42.44 (19)
N1A—C18A—C19A—C24A	−148.34 (13)	N1B—C18B—C19B—C24B	142.42 (12)
O4A—C18A—C19A—C20A	−143.71 (14)	O4B—C18B—C19B—C20B	132.94 (14)
N1A—C18A—C19A—C20A	33.72 (18)	N1B—C18B—C19B—C20B	−42.21 (17)
C24A—C19A—C20A—C21A	1.6 (2)	C24B—C19B—C20B—C21B	−0.3 (2)
C18A—C19A—C20A—C21A	179.56 (14)	C18B—C19B—C20B—C21B	−175.65 (13)
C19A—C20A—C21A—C22A	0.7 (3)	C19B—C20B—C21B—C22B	−0.9 (2)
C20A—C21A—C22A—C23A	−2.0 (3)	C20B—C21B—C22B—C23B	0.6 (2)
C21A—C22A—C23A—C24A	1.0 (3)	C21B—C22B—C23B—C24B	0.9 (2)
C20A—C19A—C24A—C23A	−2.6 (2)	C20B—C19B—C24B—C23B	1.8 (2)
C18A—C19A—C24A—C23A	179.40 (14)	C18B—C19B—C24B—C23B	177.18 (13)
C22A—C23A—C24A—C19A	1.3 (3)	C22B—C23B—C24B—C19B	−2.0 (2)

### Hydrogen-bond geometry (Å, º)

**Table:**

*D*—H···*A*	*D*—H	H···*A*	*D*···*A*	*D*—H···*A*
C21*A*—H21*A*···O2*B*^i^	0.95	2.41	3.1270 (19)	132
C14*B*—H14*B*···O3*A*^ii^	0.95	2.50	3.4345 (17)	168
C15*B*—H15*B*···O4*A*^iii^	0.95	2.62	3.4638 (17)	148
C22*B*—H22*B*···O2*A*	0.95	2.54	3.2054 (17)	127
C14*A*—H14*A*···O3*B*	0.95	2.44	3.2303 (16)	140

Symmetry codes: (i) *x*, *y*+1, *z*; (ii) *x*, *y*−1, *z*; (iii) −*x*+1, −*y*+1, −*z*+2.
